# Reduced-Fat Response of *Lactobacillus casei* subsp. *casei* SY13 on a Time and Dose-Dependent Model

**DOI:** 10.3389/fmicb.2018.03200

**Published:** 2018-12-21

**Authors:** Zhenhu Jia, Xiaoyang Pang, Jiaping Lv

**Affiliations:** ^1^Institute of Food Science and Technology, Chinese Academy of Agricultural Sciences, Beijing, China; ^2^College of Life Science, Shanxi Normal University, Linfen, China; ^3^Beijing Advanced Innovation Center for Food Nutrition and Human Health, Beijing Technology and Business University, Beijing, China

**Keywords:** *Lc* SY13, reduced-fat, intestinal mucosal, high-fat, Syrian golden hamsters

## Abstract

A reduced-fat effect of probiotics was primarily derived functionally rather than structurally, and we investigated the ultra-structural aspect of the gut mucosa in Syrian golden hamsters with high-fat diet by feeding with *Lactobacillus casei* subsp. *casei* SY13 (*Lc* SY13). 36 adult-male Syrian golden hamsters were randomly grouped into four; control group (G1), high-fat group (G2), high-dose group (G3), and low-dose group (G4). The G1 hamsters were fed a standard normal chow diet, while those in other groups were fed a high-fat chow diet for duration of 8 weeks. With the use of oral gavage, G1 hamsters were administered 1 mL of skim milk/hamster/day, while G3 and G4 hamsters were administered *Lc* SY13 at 4.1 × 10^10^ or 4.1 × 10^8^ cells/hamster/day. At 14, 28, and 56 days consecutively, three golden hamsters from each group were sacrificed by carotid, taking blood from eyeball for quantitative detection of hamsters serum total cholesterol (TC), triacylglycerol (TG), high-density lipoprotein (HDL), and low-density lipoprotein (LDL). At 56 days, Taqman-MGB fluorescence probe was used for the quantitative detection of *Lc* SY13 in the intestinal mucosal, and their ileum was viewed using transmission electron microscopy (TEM). Screening of the ileum microvilli of the hamsters showed that at 56 days, G3 was significantly (*P* < 0.05) bigger than other groups while its serum TC, TG, and TDL decreased. *Lc* SY13 was detected in the intestines, and was significantly (*P* < 0.05) higher in the ileum of G3 than those of G4. In conclusion, *Lc* SY13 may play a remarkable reduced-fat response role by improving high-fat uptake as well as its metabolism and transport; most especially in G3. The reduced-fat response of the *Lc* SY13 differed in a time and dose-dependent manner. These findings indicated that probiotic strains of *Lc* SY13 can reduce fat level, thus suggesting its potential in ameliorating obesity-related diseases.

## Introduction

The benefits of probiotics have been recognized and explored for over a century. The effect of probiotics and their interactions with their hosts have gradually been realized around the world (Salminen et al., 2009). Some data suggests that probiotics are beneficial for various illnesses, such as enteropathy, inflammation, immunological diseases or metabolic syndromes. They have recently been used for preventing obesity (Bubnov et al., 2015; Bernini et al., 2016; Nova et al., 2016). In China, as a result of the dramatic economic development and associated improvements in diet (especially high-fat diet), the prevalence of obesity has undergone significant increase in the last two decades. Obesity is a widely known key risk-factor for many diseases (Han and Lean, 2016). As probiotic supplements can affect host nutritional metabolism, which affects energy storage, adiposity, and nutrient absorption (An et al., 2011), supplementing the diet with probiotics may be an alternative strategy for combating obesity and related disorders (Novotny Nunez et al., 2015). Usman and Hosono (1999) indicated the capability to remove cholesterol from a culture medium using strains of *Lactobacillus gasseri*. Portugal et al. (2006) suggested that the effect of *L. delbrueckii* on cholesterol metabolism was through ApoE. These health-promoting effects may be related to the anti-obesity effects of lactic acid bacteria. With enough clinical evidence to support certain health claims attributed to selected probiotics (Deng et al., 2013), more proven mechanisms of action of probiotics have recently been published (Xie et al., 2016). However, substantial work needs to be done to further improve their beneficial functions such as the protection of intestinal mucosal. Although some studies have examined the anatomical development of the enteric mucosa in rats (Lutton, 1996), little is known about the gross morphological and ultra-structural adaptations that occur in the enteric mucosa of rats with high-fat diets.

*Lactobacillus casei* subsp. *casei* SY13 (*Lc* SY13) isolated from the traditional fermented dairy products, is an excellent probiotic microorganism in ameliorating enteric diseases and maintaining health (Zhang et al., 2009). Syrian golden hamsters has been previously found to be suitable models for the evaluation of human fat-induced and diet-induced diseases (Jiang et al., 2013). The present study provides detailed illustrations of the microanatomical and ultra-structural adaptations that occur in the intestinal mucosa of Syrian golden hamster after oral gavage administration of *Lc* SY13.

## Materials and Methods

### Bacterial Strain

*Lactobacillus casei* subsp. *casei* SY13 strains used for this study was previously maintained at -80°C in MRS broth (Land Bridge, Beijing, LB) with 25% (v/v)glycerol (Sigma-Aldrich, St. Louis, MO, United States). Working cultures were prepared from frozen stocks by two sequential transfers into MRS broth and incubations were conducted sequentially at 37°C for 24 and 18 h.

### Preparation of Bacteria Suspensions

*Lactobacillus casei* subsp. *casei* SY13 suspensions were prepared by suspending lyophilized bacteria powered into skim milk as its substrate. Colony counts was conducted prior to administration into hamsters, in order to ensure the surviving bacteria counts at 4.1 × 1010 cfu/mL or 4.1 × 108 cfu/mL.

### Experimental Design

#### Ethics Statement

The experiments were approved by the Research Ethics Committee of the Chinese Academy of Medical Sciences and Peking Union Medical College (approval number: SCXK (Jing) 2014-0008). The animals were maintained, and the experiments were conducted in accordance with the Institutional Animal Care and Use Committee Acts of Chinese Academy of Medical Sciences and Peking Union Medical College, using the 1996 Guide for the Care and Use of Laboratory Animals (Institute of Laboratory Animal Resources on Life Sciences, National Research Council, National Academy of Sciences, Washington, DC, United States).

#### Animals and Experimental Protocol

Fourteen-weeks-old Syrian golden hamsters were obtained from Vital River Laboratory Animal Technology Co., Ltd. (Beijing, China). Throughout the acclimatization and study periods, all animals had *ad libitum* access to food and water and were maintained on a 12 h light/dark cycle in a facility with an ambient temperature of 21 ± 2°C, and a relative humidity of 45 ± 10%. Thirty-six adult male Syrian golden hamsters (160 ± 180 g) were acclimatized for 7 days in cages prior to the initiation of the study. The hamsters were then randomly allocated into four groups; control group (G1), high-fat group (G2), high-dose group (G3), and low-dose group (G4). G1 hamsters were fed a standard normal chow diet, while those in other groups were fed a high-fat chow diet (HFK Bioscience Co., LTD., Beijing, China) daily for 8 weeks. At the same time, G1 hamsters were administered by oral gavage, 1 mL of skim milk/hamsters/day, while those in high and low dose groups were administered *Lc* SY13, also by oral gavage at 4.1 × 10^10^ or 4.1 × 10^8^ cells/hamsters/day. The standard normal chow diet contains of 98% standard diet and 2% fat, while the high-fat chow diet contains 85.2% standard diet, 14.5% fat, and 0.3% cholesterol.

#### Analysis of Serum TC, TG, HDL, and LDL

The serums were collected after hamsters were killed. The serum TC, TG, HDL, and LDL were analyzed in triplicates using ELISA kit (Shanghai Elisa Biotech Inc., China).

#### DNA Extraction

The contents in 1 cm jejunum, ileum, cecum, and colon of hamsters were separately collected, and the total DNA of intestinal microorganism was extracted by TIANamp Stool DNA Kit (DP328, TIANGEN). The specific steps were operated according to the instructions, and the extracted DNA was stored at -80°C.

#### Detection of *Lactobacillus casei* SY13 in the Intestines by Taqman – MGB Probe

The total DNA of microorganisms identified in the jejunum, ileum, cecum, and colon contents of each hamsters, were analyzed using probe Taqman – MGB (FAMCTCAAAAATGGATCTTGMGB) with real time fluorescence quantitative PCR detection. The total volume of the reaction system was 20 μL, containing 1 μL 06232F (10 μm), 1 μL 06232R (10 μm), 1 μL 06232P (10 μm), 1 μL template DNA, 10 1 μL TaqMan^®^Universal PCR Master Mix, and 6 μL sterilized ultra-pure water. The conditions of fluorescence quantitative PCR reaction at a total of 40 cycles comprised of 50°C enzyme activation for 2 min, 95°C pre-denaturation for 10 min, circulation stage, 95°C denaturation for 15 s, and 58°C annealing and extension for 60 s. Fluorescence detection was set during annealing and extension stage.

#### Specimen Preparation for Transmission Electron Microscopy

From each group of hamster, three were sacrificed by Carotid at 14, 28, and 56 days consecutively. Immediately after being sacrificed, the middle part of the ileum (near the ileo-cecal colon) was taken and flushed with phosphate-buffered saline. Approximately 2 cm length of flushed ileum was fixed in a mixed fixative solution of 3% glutaraldehyde and 4% paraformaldehyde in 0.1 M Cacodylate buffer (pH 7.4). Tissue specimens were post-fixed with 1% Osmium tetroxide in the same ice-cold buffer for 2 h. They were then stained overnight with 0.5% uranyl acetate *en bloc*, and embedded in Spur’s plastic mixture. Silver to gray ultra-thin sections were cut, stained with lead nitrate, and then examined under a Hitachi H-7100 transmission electron microscope (Hitachi, Ibaragi) at 75 kV (Yamauchi and Snel, 2000).

#### Data Analysis

The data were statistically analyzed with SPSS 20.0 software package, and the data were expressed in x ± s, and *P* < 0.05 was statistically significant.

## Results

### Serum TC, TG, HDL, and LDL Levels During Diet Administration

As shown in Table 1, the serum TC, TG and LDL levels in G1 hamsters were significantly lower than those of other groups (*P* < 0.05). The serum HDL level of G2 hamsters was significantly (*P* < 0.05) higher than those of G1 and G3 hamsters. The results also showed that serum HDL levels of G3 hamsters was lower than those of G2 and G4 hamsters, but significantly (*P* < 0.05) higher than that of the G1 hamsters. The results in Table 2 showed that serum TC, TG, and LDL levels in G1 hamsters were significantly lowered than those of the other groups at 28 days (*P* < 0.05). However, the serum HDL level of G2 hamsters was significantly higher than those of the G1 and G3 (*P* < 0.05); while the serum HDL level of G3 hamster was lower than those of the G2 and G4, though higher than that of the G1 (*P* < 0.05).

**Table 1:** Serum TC, TG, HDL, and LDL levels at the 14 days (pg/ml and x ± SD).

Group	TC	TG	HDL	LDL
G1	3.75 ± 0.58^a^	1.64 ± 0.31^a^	1.75 ± 0.15^a^	1.69 ± 0.62^a^
G2	9.45 ± 2.74^b^	2.88 ± 1.20^b^	3.00 ± 0.46^b^	5.24 ± 1.97^b^
G3	9.92 ± 4.00^b^	4.14 ± 1.89^b^	2.41 ± 0.26^c^	5.69 ± 2.02^b^
G4	9.35 ± 1.47^b^	3.58 ± 1.82^b^	2.74 ± 0.25^bd^	5.17 ± 0.98^b^


*The superscript represents the results of each column, and the same letters stands for not significant differences.*

**Table 2:** Serum TC, TG, HDL, and LDL levels at the 28 days (pg/ml and x ± SD).

Group	TC	TG	HDL	LDL
G1	4.41 ± 0.83^a^	1.48 ± 0.90^a^	1.88 ± 0.12^a^	2.25 ± 0.82^a^
G2	14.17 ± 5.47^b^	3.33 ± 3.23^b^	3.37 ± 0.38^b^	8.46 ± 3.44^b^
G3	11.74 ± 5.58^b^	3.68 ± 2.71^b^	2.96 ± 0.37^c^	6.98 ± 3.61^b^
G4	11.54 ± 1.27^b^	2.79 ± 1.17^b^	3.32 ± 0.28^bd^	6.71 ± 1.22^b^


*The superscript represents the results of each column, and the same letters stands for not significant differences.*

As shown in Table 3, the serum TC, TG, HDL, and LDL levels in G1 hamsters were significantly lower than those of the other groups (*P* < 0.05) at 56 days. The hamsters serum TC, TG, and LDL content of G2 was significantly higher than that of the G1 and G3 (*P* < 0.05); The hamsters serum TC, TG, and LDL content of G3 was lower than that of the G2 and G4, but higher than that of the G1 (*P* < 0.05).

**Table 3:** Serum TC, TG, HDL, and LDL levels at the 56 days (pg/ml and x ± SD).

Group	TC	TG	HDL	LDL
G1	4.71 ± 0.81^a^	2.53 ± 1.18^a^	1.89 ± 0.14^a^	2.20 ± 0.69^a^
G2	18.16 ± 5.36^b^	7.33 ± 5.05^b^	2.80 ± 0.44^b^	12.06 ± 4.29^b^
G3	13.52 ± 3.38^c^	4.00 ± 1.95^c^	2.91 ± 0.27^b^	8.25 ± 2.62^c^
G4	15.26 ± 5.36^bc^	5.79 ± 4.08^bc^	2.68 ± 0.38^b^	9.07 ± 4.08^bc^


*The superscript represents the results of each column, and the same letters stands for not significant differences.*

### Detection of Intestinal *Lc* SY13 in Mice at 56 days

The jejunum, ileum, caecum, and colon of groups G3 and G4 hamsters were collected after sacrificing them at 56 days; to quantitatively detect the total DNA of *Lc* SY13 using Fluorescence quantitative detector. As shown in Table 4, the quantity of *Lc* SY13 in the ileum of G4 hamsters was significantly lower than those in G3 hamsters (*P* < 0.05), while the quantity of *Lc* SY13 in the cecum was significantly higher than those in G3 hamsters (*P* < 0.05).

**Table 4:** The number and distribution of *Lactobacillus casei* SY13 in the intestinal tract of mice (x ± SD).

Groups	Jejunum	Ileum	Cecum	Colon
	**56 days**			
	
G1	–	–	–	–
G2	–	–	–	–
G3	25.32 ± 1.092^a^	29.45 ± 1.331^a^	22.62 ± 0.831^a^	22.33 ± 0.845^a^
G4	26.89 ± 0.862^a^	25.93 ± 1.167^b^	24.06 ± 0.780^b^	23.77 ± 0.891^a^


*The superscript represents the results of each column, and the same letters stands for not significant differences.*

### TEM Analysis of Ileum Cell at 14 days

**FIGURE 1:**
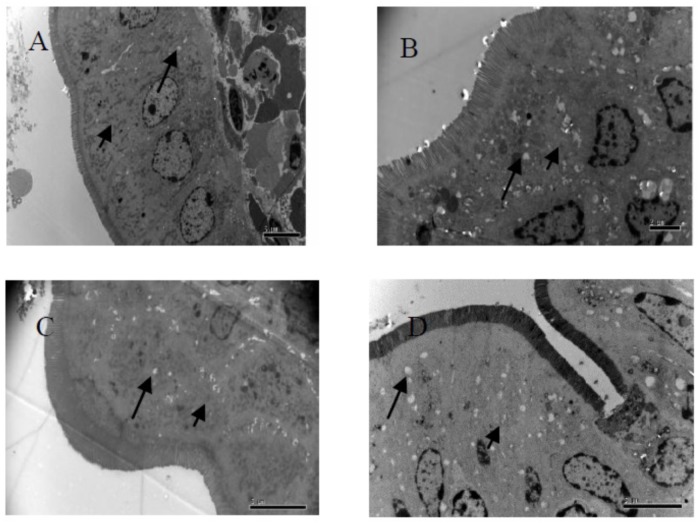
Fine structural alterations of ileal epithelial cells on the 14 days. **(A)** Control group (magnification, ×5000, Bar, 5 μm), **(B)** high fat group (magnification, ×8000, Bar, 2 μm), **(C)** higher dose group (magnification, ×6000, Bar, 5 μm), and **(D)** lower dose group (magnification, ×6000, Bar, 5 μm).

As shown in Table 5 and Figure 1, the density of microvilli on the ileum of G1 hamsters was significantly looser than those of the other groups (*P* < 0.05). The height of microvilli on the ileum of G1 hamsters was significantly shorter than those of the other groups (*P* < 0.05). There were many fat droplets (long arrow as shown in Figures 1B–D) in the epithelial cells and intercellular space. Also, there were many aggregated mitochondria mainly distributed in the cytoplasm near the nucleus in all groups. The number of mitochondria was increased in all groups (short arrow shown in Figure 1).

**Table 5:** The morphology and density of microvilli of hamster’s ileum at the 14 days (μm, log10/μm^2^, and x ± SD).

Group	Width	Height	Density
G1	0.15 ± 0.79^a^	0.93 ± 0.31^a^	4.94 ± 0.54^a^
G2	0.09 ± 0.18^b^	1.46 ± 0.38^b^	6.47 ± 0.67^b^
G3	0.12 ± 0.34^a^	1.64 ± 0.15^b^	8.92 ± 0.67^b^
G4	0.08 ± 0.29^b^	1.28 ± 0.47^c^	7.12 ± 0.13^b^


*The superscript represents the results of each column, and the same letters stands for not significant differences.*

### TEM Analysis of Ileum Cell at 28 days

**FIGURE 2:**
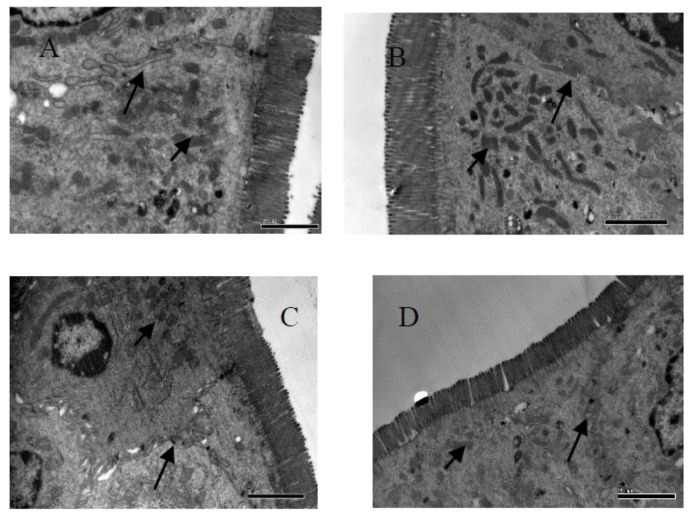
Fine structural alterations of ileal epithelial cells on the 28 days. **(A)** Control group (magnification, ×15,000, Bar, 2 μm), **(B)** high fat group (magnification, ×15,000, Bar, 2 μm), **(C)** higher dose group (magnification, ×15,000, Bar, 2 μm), and **(D)** lower dose group (magnification, ×15,000, Bar, 2 μm).

**FIGURE 3:**
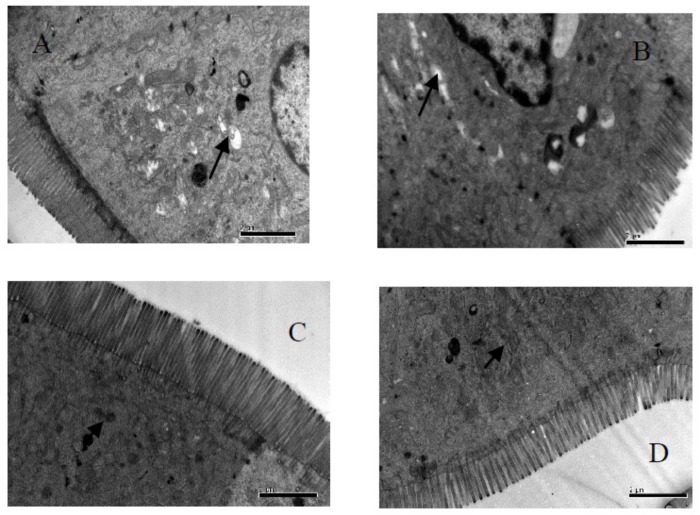
Fine structural alterations of ileal epithelial cells on the 56 days. **(A)** Control group (magnification, ×15,000, Bar, 2 μm), **(B)** high fat group (magnification, ×15,000, Bar, 2 μm), **(C)** higher dose group (magnification, ×15,000, Bar, 2 μm), and **(D)** lower dose group (magnification, ×15,000, Bar, 2 μm).

The height of microvilli on the ileum of G1 hamsters was significantly (*P* < 0.05) shorter than those of the other groups (Table 6 and Figure 2). However, the density of microvilli on the ileum of G1, G2, and G4 were hamsters significantly looser than that in G3 hamsters (*P* < 0.05). In ileal epithelial cells, the number of mitochondria was increased in all groups (short arrow shown in Figure 3). More desmosomes were found in all groups (long arrow shown in Figure 3). These desmosomes, embedded in the cytoplasm appeared in the adjacent cells and had uneven digitatione-like structures.

**Table 6:** The morphology and density of microvilli of hamster’s ileum at the 28 days (μm, log10/μm^2^, and x ± SD).

Group	Width	Height	Density
G1	0.05 ± 0.14^a^	0.93 ± 0.06^a^	7.40 ± 1.00^a^
G2	0.04 ± 0.11^a^	1.50 ± 0.31^b^	11.91 ± 1.10^b^
G3	0.07 ± 0.17^b^	1.93 ± 0.11b^c^	14.20 ± 1.57^c^
G4	0.05 ± 0.35^a^	1.47 ± 0.21^b^	11.80 ± 0.73^b^


*The superscript represents the results of each column, and the same letters stands for not significant differences.*

### TEM Analysis of Ileum Cell at 56 days

Our results in Table 7 and Figure 3, showed the density of microvilli on the ileum of G1 hamsters, and was significantly looser than those of the other groups (*P* < 0.05). The height of microvilli on the ileum of G1 hamsters was significantly shorter than that of G2 and G4 (*P* < 0.05). The density of microvilli on the ileum of G1, G2, and G4 hamsters were significantly looser than that in G3 (*P* < 0.05). The number of mitochondria increased in all groups (short arrow shown in Figure 3). The microvilli in G3 (Figure 3C) were apparently longer and neater than those in other groups (Figures 3A,B,D). Big fat droplets were found in G1 and G2 (long arrow shown in Figures 3A,B). In comparism to G1 and G2, the microvilliin G3 and G4 were apparently longer and denser, especially in G3.

**Table 7:** The morphology and density of microvilli of hamster’s ileum at the 56 days (μm, log10/μm^2^, and x ± SD).

Group	Width	Height	Density
G1	0.14 ± 0.29^a^	1.15 ± 0.68^a^	7.24 ± 0.31^a^
G2	0.16 ± 0.14^a^	1.53 ± 0.13^b^	11.49 ± 0.17^b^
G3	0.11 ± 0.49^b^	1.94 ± 0.28^c^	14.00 ± 0.33^c^
G4	0.15 ± 0.18^a^	1.45 ± 0.12^a^	12.27 ± 0.57^b^


*The superscript represents the results of each column, and the same letters stands for not significant differences.*

## Discussion

This study was aimed at verifying the reduced-fat effect of the *Lc* SY13 at a micro-structural level. *Lc* SY13 has been previously proven to be appropriate to ameliorate enteric diseases, as well as maintaining health. Thus in this paper, we presented a direct demonstration to further verify the fat-reducing properties of *Lc* SY13 by oral administration at 4.1 × 10^10^ or 4.1 × 10^8^ cells/hamsters/day. Due to the established fact that hamsters are endowed with cholesterol ester transfer protein and all of the enzymatic pathways in lipoproteins and bile metabolism; their response to a high-fat diet are similar to humans (Stancu et al., 2014). Thus, we chose the fourteen-week-old Syrian golden hamsters (160 ± 180 g) as our feeding animals. When the hamsters fed high-fat chow were treated with *Lc* SY13 under high-fat circumstances, intestinal microbiota and the topography of enteric mucosa could easily be altered (Zhang and Yang, 2016). The study established a temporary relationship between *Lc* SY13 and intestinal absorption functions in the small intestine of rodent high-fat diet-induced obesity.

Because the mammalian intestine is an important digestive organ and the intestinal microbiota plays a crucial role in the development of intestinal mucosal (Heinritz et al., 2016), stimulation of mucosal tissue by high-fat and *Lc* SY13 consequently affected the intestinal mucosal cells. Since microvilli can make epithelial surface area increase 20–30 times under high-fat circumstances, the intestinal mucosal of hamsters fed high-fat chow can promote its fat absorption. Increased number of mitochondria may also provide its growing requirement of material and energy. More desmosomes indicated that the intestinal mucosal of the hamster increased its cell membrane areas and the cell communication, which may be a part of a compensatory mechanism in improving fat absorption (Brooke et al., 2012). At 28 and 56 days of our study, the microvilli of the mucosal of G3 hamsters were apparently longer and neater than those in other groups. These results showed that the *Lc* SY13 accommodated the hamsters’ intestine mucosal and mediated the ingestion of fat-rich diets until the 28 days. As an important modulator of intestinal microbiota, the *Lc* SY13 may play a critical role by promoting the high-fat uptake in small intestinal.

Though the real-time quantitative PCR (qPCR) with a TaqMan^®^minor-groove-binder (MGB) probe often was used to detect viruses (Yang et al., 2018), it was also reported to detect some lactic acid bacteria in foods (Mackay, 2004; Grattepanche et al., 2005). In this study, based on the special sequence fragments of *Lc* SY13 DNA, an MGB probe set was designed to detect the intestinal *Lc* SY13 by using gradually diluted intestinal content DNA as templates (Jia et al., 2017). At 56 days, the number of *Lc* SY13 of hamsters ileum in G4 hamsters was significantly lower than that in G3, while the G1 or G2 failed to detect the *Lc* SY13. Our results were consistent with the description of G. Saxami, that the distribution of *L. casei ATCC393* in the intestinal tract, and the strain in the jejunum and ileum are relatively high (Saxami et al., 2012). This may be associated with *Lc* SY13 which invades the intestine for a long time, causing the microenvironment of the intestinal tract to be beneficial to the growth and transformation of *Lc* SY13. The long time intragastric administration made with *Lc* SY13 reached a balanced state with other bacteria in the intestinal micro *biogeocenose*, which is consistent with the reports of other lactic acid bacteria colonization in the intestine (Matsuki et al., 2013; Mohania et al., 2014). These results showed that the *Lc* SY13 had a high ability to survive under gastrointestinal tract conditions and to adhere to intestinal cells. The qPCR developed in this study provides a rapid, reliable, and sensitive diagnostic assay for the detection of *Lc* SY13, and it could be applied to other diagnostic procedures for the probiotics.

High blood TC and TG levels are commonly considered import modulators and biomarkers of obesity processes (Tsai et al., 2009). Therefore, the management of these two parameters is necessary for preventing obesity. At 56 days, the hamster in G3 by oral administration of *Lc* SY13, was found to significantly reduce TC, TG, and LDL levels. *Lc* SY13 may have lipid-lowering actions by decreasing serum TC and TG. As the *Lc* SY13 may play a critical role by promoting the high-fat uptake in small intestinal at 56 days, *Lc* SY13 were detected in the cecum and colon of G3 and G4. Their possible mechanism is the promotion of lipid degradation and transport in large intestinal, as well as deconjugation of bile salts by bile-salt hydrolase (BSH) assimilation of TC into bacterial cell membranes to reduce TC level (Kim, 2014; Tsai et al., 2014). The *Lc* SY13 could remove lipid droplet via binding onto the bacterial cellular surface and assimilate cholesterol in large intestinal. Moreover, the *Lc* SY13 could release some metabolic material that affects the lipid metabolism of hamsters such as propionic acid and butyric acid (Bosch et al., 2012).

All results in this study, showed that *Lc* SY13 have potential beneficial effects in reduced-fat responses. Their possible mechanism may promote the high-fat uptake in small intestinal mucosa by adhering to intestinal cells, and removing lipid droplets via binding onto the bacterial cellular surface and assimilation of cholesterol in large intestinal mucosa, thus releasing some metabolic materials. However, these mechanisms depends on the dose of the *Lc* SY13 by oral gavage, as well as the time at which *Lc* SY13 reached a balanced state with other bacteria in the intestinal micro *biogeocenose*.

## Conclusion

Our study provides ultra-structural evidences to support our hypothesis that *Lc* SY13 may have potential beneficial effects as a therapy for reducing fat levels. The reduced-fat response of *Lc* SY13 in hamsters differs in a time and dose-dependent manner in the intestinal mucosa. Combined, these characteristics suggest that *Lc* SY13 could be excellent candidates for reducing high blood cholesterol levels. Further studies are needed to elucidate the reduced-fat effects of *Lc* SY13, with a longer period of supplementation.

## Author Contributions

XP and JL contributed in study conception and experimental design. ZJ carried out specific experiments. XP and ZJ wrote the manuscript. All authors have read and approved of the manuscript.

## Conflict of Interest Statement

The authors declare that the research was conducted in the absence of any commercial or financial relationships that could be construed as a potential conflict of interest.
